# Tackling the Problem of Tendon Adhesions: Physical Barriers Prepared from α-Amino Acid-Based Poly(ester amide)s

**DOI:** 10.3390/polym17030395

**Published:** 2025-02-01

**Authors:** Sofia Saraiva, Francisca Rénio, Patrícia Pereira, Patrícia Santos, Carlos T. B. Paula, Amílcar Ramalho, Arménio C. Serra, Ana C. Fonseca

**Affiliations:** 1 Centre for Mechanical Engineering, Materials and Processes (CEMMPRE), ARISE, Department of Chemical Engineering, University of Coimbra, Pólo II, Rua Sílvio Lima, 3030-790 Coimbra, Portugal; uc44571@uc.pt (S.S.); 2021201023@student.uc.pt (F.R.); papereira@ipn.pt (P.P.); uc201345659@student.uc.pt (P.S.); ctadeu@eq.uc.pt (C.T.B.P.); aserra@eq.uc.pt (A.C.S.); 2 IPN, Instituto Pedro Nunes, Associação para a Inovação e Desenvolvimento em Ciência e Tecnologia, Rua Pedro Nunes, 3030-199 Coimbra, Portugal; 3 Centre for Mechanical Engineering, Materials and Processes (CEMMPRE), ARISE, Department of Mechanical Engineering, University of Coimbra, Pólo II, Rua Luís Reis Santos, 3030-788 Coimbra, Portugal; amilcar.ramalho@dem.uc.pt

**Keywords:** poly(ester amide)s, α-amino acids, electrospinning, tendon adhesions

## Abstract

In this work, electrospun membranes of α-amino acid based poly(ester amide)s (AAA-PEAs) from L-alanine (PEA_ala) or L-phenylalanine (PEA_phe) were successfully prepared to be used as physical barriers in the orthopedic field. Also, blends of these two polymers were used in different weight ratios (25:75, 50:50 and 75:25) to obtain physical barriers with different properties. All membranes had a suitable pore size to prevent fibroblast infiltration, and their porosity and permeability values were in a range that allowed the passage of nutrients. The membrane made from a blend of 25%wt of PEA_ala and 75% wt of PEA_phe showed the highest value of swelling capacity, suggesting a higher lubricant feature. The same membrane suffered a more pronounced degradation, as evidenced by the in vitro enzymatic degradation tests. All membranes showed suitable toughness values, a crucial property with regard to application. In vitro cytotoxicity tests performed with a NIH3T3 fibroblast cell line revealed decreased cell viability after 7 days, suggesting that these membranes are not ideal substrates to promote fibroblast adhesion and proliferation. These membranes as physical barriers represent a significant advance in the field given the limited literature on electrospun AAA-PEAs and their use to prevent tendon adhesion.

## 1. Introduction

Tendons are responsible for converting muscle contraction into joint movement. Tendon lesions are very common and affect 6–7 million people worldwide [1]. Intrasynovial tendons, of which the flexor tendons are an example, are the most likely to be affected by injuries. These tendons are surrounded by a thin layer known as the tendon sheath. This sheath consists of an inner membrane, the synovial membrane, and an outer membrane, the fibrous membrane, which are responsible for the nutrition and protection of the tendon [2,3,4]. Between these membranes circulates the synovial fluid, which consists mainly of hyaluronic acid (HA), responsible for the movement and lubrication of the tendons [5]. When a lesion occurs, this sheath is damaged, compromising tendon healing but, more importantly, allowing the proliferation of exogenous fibroblasts, which are recruited during the healing process, leading to the formation of fibrous bridges between the tendon and the surroundings, culminating in the formation of tendon adhesions [6,7]. Adhesions following tendon lesions affect 30–40% of patients [8] and are a significant problem as they can lead to restricted movement and pain [9,10].

Over the years, various strategies to prevent tendon adhesions have been studied and tested, in particular the use of physical barriers [11,12,13]. Membranes appear to be the most promising material for the development of physical barriers, but they should have several properties to be effective. They should prevent the passage of exogenous fibroblasts while allowing the passage of nutrients that are important for the restoration of normal tendon function [8,14]. The material should be easy to apply and manipulate during surgical procedures [3,7]. Furthermore, it must not hinder the natural movement of the tendon. A lubricant surface would be optimal to minimize friction and optimize tendon gliding [13]. Also, considering the main stages of tendon healing where adhesions are most likely to occur, the material must maintain its integrity for at least 6 weeks.

Electrospinning (ES) has been the most commonly used technique for the development of fibrous membranes with the potential to solve this problem [4,15,16,17,18,19,20,21]. This process can be used to produce membranes with a large surface area [15,22] consisting of nanofibers with diameters in the same range as the structural proteins of the extracellular matrix (ECM) [23]. Moreover, it is possible to customize the pore size and porosity according to the requirements of each specific application. In addition, the ES allows for the easy adjustment of mechanical properties. These properties are particularly relevant since the physical barriers should be able to withstand the forces to which the tendon is subjected and must be able to be easily handled without rupture.

Although many synthetic and natural polymers have been tested to prepare these membranes using ES, none of the solutions have been able to fulfil the properties required to completely prevent the formation of tendon adhesions [15,16,24,25,26]. Polyesters, such as poly(lactic acid-*co*-glycolic acid) (PLGA) and poly(*ε*-caprolactone) (PCL), are the most commonly used synthetic polymers. However, these polyesters are known to release some acidic byproducts during their degradation, leading to acidification of the surrounding tissues, which increases the inflammatory process and might exacerbate the occurrence of tendon adhesions [24]. Natural polymers, such as HA, can also be used in the preparation of these membranes, but they do not have the necessary mechanical properties, so they are usually used in combination with synthetic polymers.

Therefore, there is a clinical need for the development of new materials that can effectively prevent these adhesions, have the necessary mechanical properties, and avoid the release of acidic degradation byproducts.

Poly(ester amide)s (PEAs), more specifically α-amino acid based poly(ester amide)s (AAA-PEAs), can be seen as promising materials for this application. AAA-PEAs combine in the same polymer ester bonds (-COO-), which confer better biodegradability, processability and solubility, and amide bonds (-NHCO-), which confer better thermal and mechanical properties. The ability to readily adjust properties and the characteristic of these polymers to release both acidic and basic products during their in vivo degradation result in a neutralization effect, thereby preventing acidification of the surrounding environment [27,28]. Recently, our research group [21] reported the use of electrospun membranes based on AAA-PEAs and PCL to be used in the prevention of tendon adhesions. The results clearly showed that the inclusion of AAA-PEAs in the membrane formulation enhanced its properties towards the envisaged application.

Therefore, in this work, novel electrospun membranes based solely on AAA-PEAs were developed to be used as potential physical barriers for the prevention of tendon adhesions. The AAA-PEAs were able to be electrospun by a conventional electrospinning process, representing a major breakthrough in the field, as typically AAA-PEAs are very hard to be electrospun. The membranes were characterized regarding their thermal, mechanical properties and cytocompatibility to assess their suitability for use in preventing tendon adhesions.

## 2. Materials and Methods

### 2.1. Materials

L-alanine, L-phenylalanine, 1,6-hexanediol, p-toluenesulfonic acid monohydrate (p-TSA), sebacoyl chloride (SC) (>95%) and *N*-hydroxysuccimide (NHS) were obtained from TCI Europe (Zwijndrecht, Belgium). José Manuel Gomes dos Santos, Lda (Odivelas, Portugal) provided the *N,N*′-dimethylformamide (DMF), acetonitrile, ethanol (96%), toluene and 2-propanol. DMF was dried over sodium sulfate and calcium chloride and distilled prior use. 2,2,2-trifluoroethanol (TFE), triethylamine (TEA), lipase and α-chymotrypsin (from bovine pancreas) were purchased from Sigma-Aldrich (St. Louis, MO, USA). Deuterated DMSO ((CD_3_)_2_SO) and deuterated chloroform (CDCl_3_) were purchased from Eurisotop (Saint-Aubin, France). The NIH 3T3 murine cell line was obtained from Sigma-Aldrich (St. Louis, MO, USA)

### 2.2. Synthesis of AAA-PEAs

#### 2.2.1. Synthesis of the Bis (α-amino Acid) Esters from L-alanine or L-phenylalanine and 1,6-Hexanediol

In a round-bottom flask equipped with Dean–Stark apparatus and a condenser, 21.38 g of L-alanine (0.24 mol) or 39.65 g of L-phenylalanine (0.24 mol), 14.18 g of 1,6-hexanediol (0.12 mol) and 45.65 g of p-TSA (0.24 mol) were placed. After the addition of 300 mL of toluene, the reaction mixture was heated until reflux. The reaction proceeded under mechanical stirring until no more water was collected (ca. 24 h) [29,30,31,32,33]. After the cooling of the reaction mixture to room temperature, the toluene was decanted. The product was recovered by recrystallization (five times) in a solution of isopropanol/ethanol (50:50 *v*/*v*) for the bis (α-amino acid) ester of L-alanine and 1,6-hexanediol and water for bis (α-amino acid) ester of L-phenylalanine and 1,6-hexanediol. After this, each product was filtered and vacuum dried at 40 °C.

Bis (α-amino acid) ester of L-alanine and 1,6-hexanediol (BAAD-ala): yield = 43%. *T*_m_ = [167–175 °C]. ^1^H NMR (400 MHz, DMSO-d_6_): 8.24 (*d*, 4H) 7.5 (*b*, 7H), 7 (*c*, 7H), 4.10 (*e*,*g*, 6H), 2.30 (*a*, 6H), 1.6 (*h*, 4H), 1.4 (*f*,*i*, 10H) (see Supplementary Information for the ^1^H NMR spectrum, Figure S1A).

Bis (α-amino acid) ester of L-phenylalanine and 1,6-hexanediol (BAAD-phe): yield: 47%. *T*_m_ = [210–213 °C]. ^1^H NMR (400 MHz, DMSO-d_6_): 8.37 (*d*, 6H), 7.50 (*b*, 2H), 7.33/7.24 (*f*, 4H), 7.13 (*c*, 2H), 4.27 (*j*, 1H), 4 (*g*, 4H), 3.14/3 (*a*, 6H), 1.39 (*h*,*i*, 8H). (See Supplementary Information for the ^1^H NMR spectrum, Figure S1B).

#### 2.2.2. Synthesis of the Sebacoyl Chloride Activated Ester

A total of 27.62 g (0.24 mol) of *N*-hydroxysuccinimide (NHS) was dissolved in 120 mL of acetonitrile. To this solution, 33.27 mL (0.24 mol) of TEA was added dropwise. Using a second flask, 25.62 mL (0.24 mol) of sebacoyl chloride (SC) was dissolved in 80 mL of acetonitrile. In an ice bath, the solution containing the SC was added at a constant feeding rate to the reaction mixture. The reaction then proceeded for 3 h in an ice bath, and after that, 300 mL of distilled water was added to the reaction mixture to end the reaction and precipitate the product. After filtration, the product was recrystallized in acetonitrile (3 times) and then filtered and dried in a vacuum oven at 40 °C [21,29,33,34]. Activated diester from sebacoyl chloride: yield = 52%. *T*_m_ = [170–175 °C]. ^1^H NMR (400 MHz, CDCl_3_): 3.30 (*a,* 4H), 2.8 (*b,* 2H), 2.65 (*c,* 2H), 1.65 (*d,* 2H), 1.30 (*e,* 2H) (see Supplementary Information for the ^1^H NMR spectrum, Figure S2).

#### 2.2.3. Synthesis of the AAA-PEAs by Solution Polycondensation

In a round bottom flask, 0.01 mol of the monomer synthetized in Section 2.2.1 (6.05 g for BAAD-ala and 7.43 g for BAAD-phe) were dissolved in 15 mL of dry DMF. Then, 2.78 mL (0.02 mol) of TEA was added dropwise, followed by 3.96 g (0.01 mol) of the monomer synthetized in Section 2.2.2 [33,35,36]. Then, the reaction was heated to 75 °C and allowed to proceed for 5 h [21,37]. After this, the product was precipitated in water and filtered, followed by a Soxhlet extraction, using ethyl acetate, to increase the purity of the AAA-PEAs based on L-alanine (PEA-ala) and L-phenylalanine (PEA-phe). Yield: 40% for PEA_ala and 59% for PEA_phe.

### 2.3. Preparation of the Membranes by Electrospinning

Polymer solutions with a final concentration of 20 or 30% (*w/v*) were prepared with the synthesized AAA-PEAs, using TFE as solvent (Table 1). The concentration of AAA-PEAs varied according to the use of a single polymer: 30% for PEA-ala or PEA_phe and 20% for the blends of the two. The final concentration of the solution was optimized for each polymer to obtain a suitable viscosity for the electrospinning process (Letter D in the Gardner viscosity scale). The solutions were electrospun using an electrospinning apparatus (E-Fiber EF100, SKE Research equipment). The solutions were pumped at a constant rate of 0.5 mL/h to a vertical steel collector located 12 cm from the tip of the syringe. An electrical potential of 15.5 kV was applied. Electrospinning was performed at 30% relative humidity and a temperature of 25–26 °C.

### 2.4. Characterization Techniques

#### 2.4.1. Chemical Structure Identification

Attenuated total reflection Fourier transform infrared (ATR-FTIR) spectroscopy was performed on a Cary 630 FTIR spectrometer. Spectra were acquired in a 600 and 4000 cm^−1^ wavenumber range, with a spectral resolution of 4 cm^−1^ and 64 accumulations.

Proton nuclear magnetic resonance (^1^H NMR) spectroscopy was performed in a Bruker Avance 400 MHz spectrometer using a 5 mm broad-band NMR probe. All spectra were acquired at 25 °C. Deuterated chloroform (CDCl_3_) and deuterated DMSO ((CD_3_)_2_SO) were used as solvents, and the following solvent peaks were used as a reference: 7.26 ppm for CDCl_3_ and 2.50 ppm for DMSO-d_6_.

#### 2.4.2. Thermal Properties

The thermal stability of the AAA-PEAs was determined by thermogravimetric analysis (TGA) using a STA 449 F3 Jupiter instrument. The analysis was carried out between 25 and 600 °C, with a heating rate of 10 °C/min, under a nitrogen atmosphere.

The thermal behavior of the AAA-PEAs was assessed by differential scanning calorimetry (DSC) using a DSC 214 Polyma equipped with an Intracooler 40 cooling unit. The samples were heated from −50 to 180 °C, cooled to −50 °C and heated again from −50 to 200 °C. The heating rate used was 5 °C/min.

#### 2.4.3. Size Exclusion Chromatography (SEC)

Molecular weight distribution was determined using a size exclusion chromatography (SEC) set-up Viscotek TDAmax equipped with a differential viscometer (DV) and right-angle laser-light scattering (RALLS, Viscotek) and refractive index (RI) detectors all from Malvern (Pennsylvania, USA). The column set consisted of a PLgel 5 µm guard column followed by one Viscotek T5000 column and one Viscotek T4000 column. A dual-piston pump was set with a flow rate of 1 mL/min. The eluent (DMF with 0.03% *w*/*v* LiBr) was previously filtered through a 0.2 µm filter. The system was also equipped with an online degasser. The tests were performed at 60 °C using an Elder CH-150 heater. Before the injection (100 µL), the samples were filtered through a polytetrafluoroethylene (PTFE) membrane with 0.2 µm pore. The system was calibrated with narrow poly(methyl methacrylate) (PMMA) standards. The molecular weight (*M*_n_ and *M*_w_) and dispersity (*Ð* = *M*_w_/*M*_n_) of the synthesized polymers were determined by conventional calibration using the OmniSEC software version: 4.6.1.354.

#### 2.4.4. Scanning Electron Microscopy (SEM)

Morphological analysis of the membranes was performed by field emission scanning electron microscopy (FESEM) using a ZEISS MERLIN Compact/VPCompact, Gemini II. The diameter of the fibers was determined by measuring 20 random fibers using Image J 1.53t software. The pore size on the surface of the membranes was also determined using the same software.

#### 2.4.5. Porosity

The porosity of the electrospun membranes was determined following Equation (1):

(1) $$P\;\mathrm{app}\;(\%)=\left(1-\frac{m}{\rho V}\right)\times 100$$

where *m* is the mass of fibrous membrane (g), *ρ* is the density of the AAA-PEA (g/cm^3^) and *V* is the volume of the mat (cm^3^) [3]. The density of AAA-PEAs was determined using a pycnometer.

#### 2.4.6. Permeability of the Membranes to Albumin

The permeability of membranes to albumin was studied using a homemade apparatus in which two glass containers were placed in contact with a membrane between them (Supplementary Information, Figure S3). The donor solution (aqueous albumin solution with a concentration of 1.4 mg/mL) was placed in one of the containers, and in the other one, only distilled water was placed. To ensure mobility between containers, the apparatus was stirred for 1 week. A negative and a positive control were prepared to confirm the viability of the results; the negative control consisted of a donor cell with a new completely sealed cap and the positive control consisted of a donor cell with a pierced cap. The albumin concentration in each container was measured using a Bradford reagent kit from Nzytech^®^ and calculated using a previously determined calibration curve (Supplementary Information, Figure S4).

#### 2.4.7. Mechanical Tests

Tensile tests on the membranes were performed on a Inspekt Solo Universal testing machine equipped with a 500 N load cell (Hegewal&Peschke). Rectangular samples with a dimension of 5 × 1 cm were tested until fracture at a rate of 5 mm/min. The Young’s modulus was determined using the slope of the initial elastic region of the curve. All of the presented values are an average of three valid tests [3].

#### 2.4.8. Thermomechanical Properties

Dynamic mechanical thermal analysis (DMTA) was carried out using a Netzsch DMA 242D under tension mode. The tests were carried out from −100 °C to 60 °C, in multifrequency mode (1, 2, 5, 10, 20 Hz), with a heating rate of 2 °C/min.

#### 2.4.9. Water Uptake Capacity

The water uptake capacity of the membranes was evaluated in phosphate-buffered saline solution (PBS, pH = 7.4, 0.01 M) at 37 °C for 72 h, and it was calculated according to Equation (2):

(2) $$\mathrm{Water}\;\mathrm{uptake}\;(\%)=\left(\frac{W_{s}-W_{d}}{W_{d}}\right)\times 100$$

where w_d_ is the initial weight of the membranes and w_s_ is the final weight of swollen membranes. The results are presented as an average of three replicates.

#### 2.4.10. In Vitro Enzymatic Degradation of the Electrospun Membranes

In vitro enzymatic degradation tests were carried out in PBS buffer (pH = 7.4, 0.01 M), containing α-chymotrypsin from bovine pancreas (0.4 mg/mL) and L-lipase (0.26 mg/mL) [38,39]. To the degradation medium, 0.02% *w*/*v* of sodium azide was also added. The test was conducted at a temperature of 37 °C for 42 days (six weeks). Approximately 4–5 mg of each electrospun membrane was immersed in 10% (*w*/*v*) PBS and incubated at 37 °C. At certain times, the membranes were removed from the medium and dried under vacuum until constant weight was reached. The medium was replaced weekly to ensure that the enzymes maintained their activity. The degree of degradation was estimated from the weight loss of the membranes, and the results are expressed as residual weight, according to Equation (3):

(3) $$\mathrm{Weight}\;\mathrm{remaining}\;(\%)=100-\left(\frac{W_{0}-W_{t}}{W_{0}}\right)\times 100$$

where w_0_ is the initial weight of the dry samples before immersion, and w_t_ is the weight of the dry sample after incubation at specific time points. The results are an average of three replicates.

### 2.5. In Vitro Biological Studies

#### 2.5.1. Cell Culture

NIH3T3 cells were cultured with DME/F-12 1:1 (1×) supplemented with 10% *v*/*v* of Fetal Bovine Serum (FBS, BioWest), 2.50 mM L-glutamine, 15 mM HEPES buffer (HyClone™) and 100 µg/mL of streptomycin/penicillin solution (Corning™) in a humidified atmosphere of 5% CO_2_ at 37 °C. The culture medium was refreshed every 2 days, and the cells were subcultured every 6 days.

#### 2.5.2. Cytocompatibility Assay

Prior to seeding the cells, electrospun membranes were cut into circular shapes with a diameter of 0.33 cm to fit the wells of a 96-well culture plate and sterilized under UV light for 30 min on each side. The samples (performed three times in triplicate) were then soaked in complete culture medium for 30 min. NIH3T3 cells at a density of 1 × 10^4^ cells/well were seeded on the surface of each mat and maintained in a humified cell culture incubator at 37 °C with 5% CO_2_ for 1, 3 and 7 days. After these time points, the samples were incubated with 10% alamarBlue^TM^ HS Cell Viability Reagent (ThermoFisher Scientific) in culture medium. After 2 h of incubation at 37 °C, 100 μL of the supernatant was transferred to a new 96-well plate, and the absorbance at 560/590 nm (excitation/emission) was determined using a microplate reader. Cells grown on tissue culture plates without membranes were used as a negative control.

#### 2.5.3. Statistical Analysis

All data were obtained from at least three parallel samples and are expressed as mean ± SD. Statistical analyses were performed using GraphPad Prism Software version 10.4.0 (GraphPad Software Inc., La Jolla, CA, USA). Two-way ANOVA (Tukey’s multiple comparisons test) was used to evaluate the statistical differences between groups, and *p* < 0.05 was considered statistically significant.

## 3. Results

In this work, novel electrospun membranes based on AAA-PEAs were developed to be used as potential physical barriers for the prevention of tendon adhesions. Two types of AAA-PEAs were used, namely PEA-ala and PEA-Phe. They were chosen because PEA-ala showed promising properties for application in blends with PCL [21], and PEA-phe was chosen because L-phenylalanine has been shown to have an effect in preventing tendon adhesions [40]. Furthermore, L-alanine and L-phenylalanine are natural amino acids present in the synovial fluid [41]. The membranes were prepared with different weight ratios of AAA-PEAs. PEA-ala and PEA-phe membranes were also prepared for comparison.

### 3.1. Synthesis of the AAA-PEAs

**Scheme 1:**
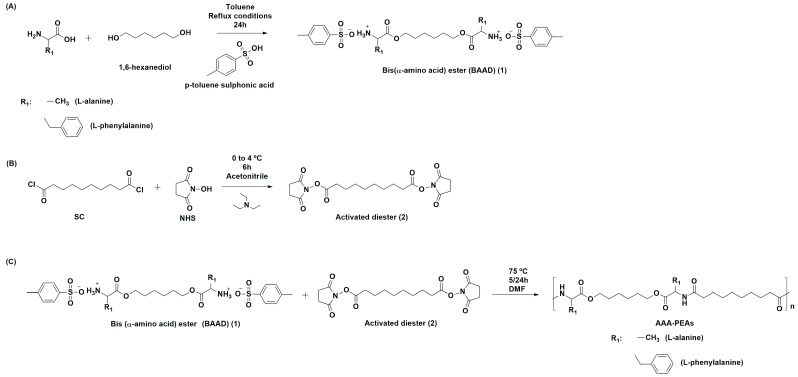
Overall synthesis strategy of the AAA-PEAs: (**A**) synthesis of the bis (α-amino acid) esters; (**B**) synthesis of the activated ester of sebacoyl chloride; (**C**) synthesis of the AAA-PEAs.

PEA_ala and PEA_phe were prepared by solution polycondensation between a diamine salt based on L-alanine or L-phenylalanine and 1,6-hexanediol and a *N*-hydroxysuccimide ester of sebacoyl chloride [33]. The reaction was carried out under basic conditions at 75 °C (Scheme 1) [33] for 5 h. A molecular weight of approximately 50 kDa (*M*_w_ = 53,140, *Ð* = 1.58) for PEA_phe and for PEA_ala (*M*_w_ = 49,266 Da, *Ð* = 1.56) was obtained. The PEA_ala_50k was not able to be electrospun and was only used in blends. This fact was attributed to the molecular weight of the PEA_ala, and thus it was decided to prepare a PEA_ala with a high molecular weight. For that, the reaction time was increased to 24 h, and for this reaction time, a PEA-ala with a molecular weight of about 100 kDa (PEA_ala_100k) was obtained (*M*_w_ = 101,854 Da, *Ð* = 1.50).

**Figure 1:**
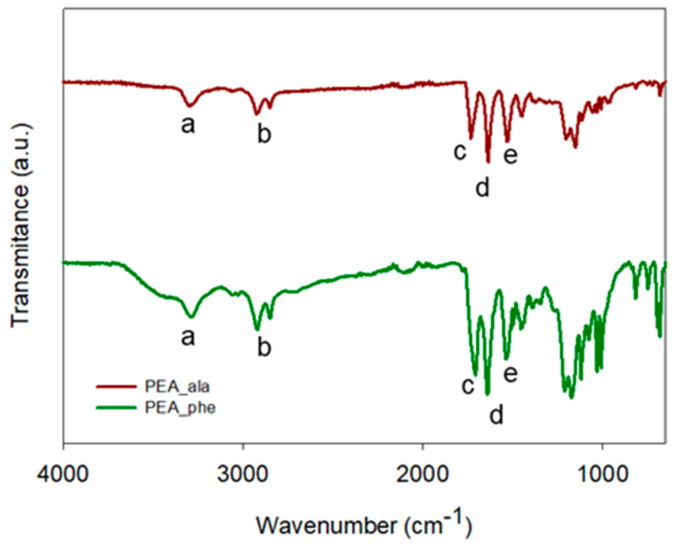
ATR FTIR spectra of PEA_ala and PEA_phe (letters a, b, c, d, and e identify the bands corresponding chemical groups belonging to the ester and amide linkages).

**Figure 2:**
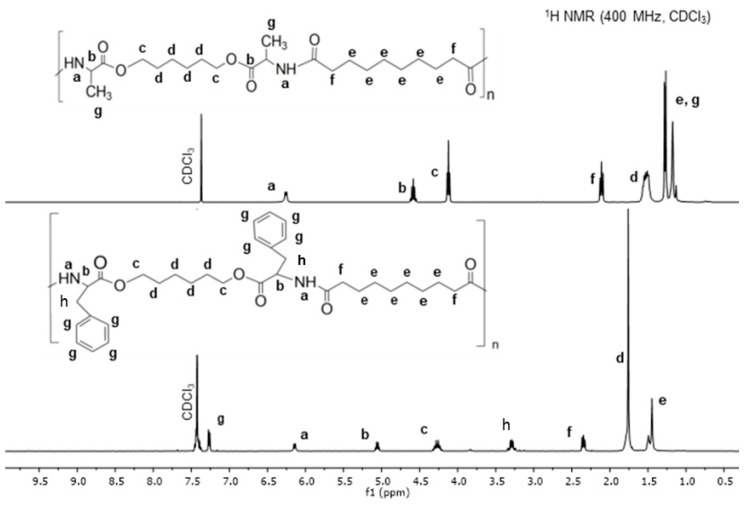
^1^H NMR in CDCl_3_ spectra of PEA-ala and PEA-Phe.

FTIR (Figure 1) and ^1^H NMR (Figure 2) spectroscopies allowed the confirmation of the chemical structure of the synthetized AAA-PEAs.

The ester linkage is represented by the stretching vibration of the -C=O group with a band at 1730 cm^−1^ (c). As for the amide group, the characteristic bands appear at 1646 cm^−1^ (d, stretching vibration of the -C=O group in the amide linkage (amide I)), at 1528 cm^−1^ (e, bending vibration of the -N-H group and stretching vibration of the -C-N group in the amide linkage (amide II)), and at 3300 cm^−1^ (a, stretching vibration of the N-H group in the amide linkage) [38,42,43].

The chemical structure of AAA-PEAs was also evaluated by ^1^H NMR spectroscopy (Figure 2). At 6.22 ppm, it is possible to see the peak assigned to the protons of the amide linkage (a). The presence of the peak (f) further confirmed the expected chemical structure confirming the success of the reaction [21,27].

#### Thermal Properties of the Synthesized Polymers

**Figure 3:**
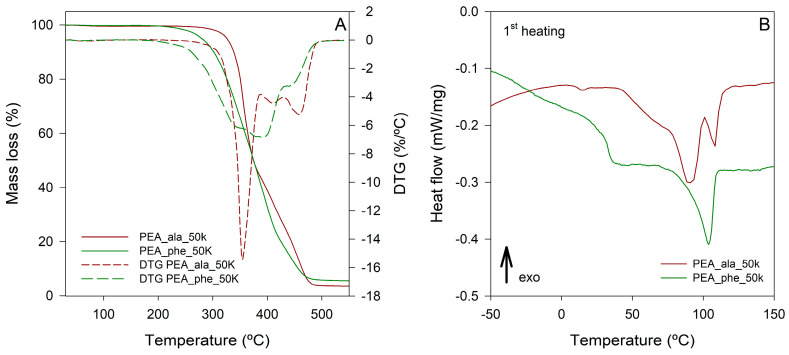
TGA thermograms of the synthetized AAA-PEAs and corresponding DTG (**A**) and heat flow curves of the same AAA-PEAs obtained by DSC in the 1st heating run (**B**).

The thermal stability of the synthesized AAA-PEAs was evaluated by TGA (Figure 3A). Below the decomposition temperature of the PEAs, their thermal behavior was studied by DSC (Figure 3B). The results obtained for PEA_ala_100k are presented in the Supplementary Information (Figure S5).

As seen in Figure 3A, the AAA-PEAs are degraded in two main stages. The first stage of weight loss can be attributed to degradation of the ester bonds and the second to the degradation of the amide bonds [21,38]. All AAA-PEAs are stable up to 280 °C, as evidenced by the *T*_5%_ and *T*_onset_ values (Table 2). This profile and values are in accordance to the reported in the literature [21,38,44].

From Figure 3B, which represents the first heating run in the DSC scans, it is possible to identify an endothermal event that can be assigned to the melting of the crystalline part of the AAA-PEAs. Therefore, the AAA-PEAs are semicrystalline after synthesis and purification. It is possible to observe that the melting transitions are broad and even present multiple melting peaks (or shoulders), indicating different crystal populations within the samples. This is a behavior typically seen for AAA-PEAs [21,43,45,46]. The temperature values (peak temperatures) at which these transitions occur are summarized in Table 2. The values obtained for PEA_ala_50k and PEA_ala_100k are quite similar despite the difference in molecular weight value. For PEA_ala_100k, the value of melting enthalpy is higher, suggesting that this AAA-PEA has more crystalline domains. The PEA_phe_50k has a similar melting enthalpy to PEA_ala_100k, which can be attributed to some interaction between the aromatic groups in the polymer structure that allows chain packing to occur [47]. For the second heating run, no endothermic event consistent with melting is observed in the heat flow curves of the AAA-PEAs (see Supplementary Information—Figure S6). This means that the AAA-PEAs cannot recrystallize from the melt and become amorphous. Most probably, the lateral groups of L-alanine and L-phenylalanine impair the polymer chains to reorganize from the melt, preventing their recrystallization [48,49]. In addition, the glass transition (*T*_g_) of the polymers was also determined by DSC (Table 2). The values obtained for *T*_g_ are in agreement with those reported in the literature for these AAA-PEAs. The PEA_ala, the one with the highest molecular weight, has a slightly higher value of T_g_, as expected. When comparing PEA_ala_50k with PEA_phe_50k, it is seen that the latter has a high *T*_g_ value, which can be ascribed to the restriction of the polymer chain movement due to the presence of a voluminous lateral group [34,41].

### 3.2. Preparation and Characterization of the Electrospun Membranes

This work aimed to develop membranes that can be used to prevent tendon adhesions with good mechanical properties to ensure easy handling and application while ensuring tendon gliding. The presence of amide groups would improve mechanical properties, and the presence of α-amino acids would make the membranes susceptible to the action of enzymes that could contribute to increased degradation and reduced immunogenic response. Indeed, several studies describing the use of polyester-based electrospun membranes as a barrier to prevent tendon adhesions show that remnants of the membrane can still be found at 6 to 8 weeks after in vivo implantation [15].

The electrospinning conditions and parameters (e.g., solution concentration, flow rate, voltage, distance between collectors, temperature, humidity) were optimized to obtain membranes free of defects (Supplementary Information, Table S1). The optimized conditions were as follows: 15.5 kV, distance of 12 cm from the collector, flow rate of 0.5 mL/h.

The AAA-PEAs were used to prepare membranes based on a single polymer (PEA_ala or PEA_phe) and membranes based on blends of the two polymers (PEA_ala/PEA_phe), with different weight ratios of AAA-PEAs (50:50; 25:75; 75:25). Electrospinning of PEA_ala_50k did not result in membranes with individualized fibers, which is why it was only used in blends with PEA_phe_50k. To obtain a membrane based on PEA_ala, the molecular weight of AAA-PEA had to be increased. A PEA_ala with a higher molecular weight (100 kDa) was synthesized, from which it was possible to produce a defect-free membrane.

#### 3.2.1. Morphology of the Membranes

**Figure 4:**
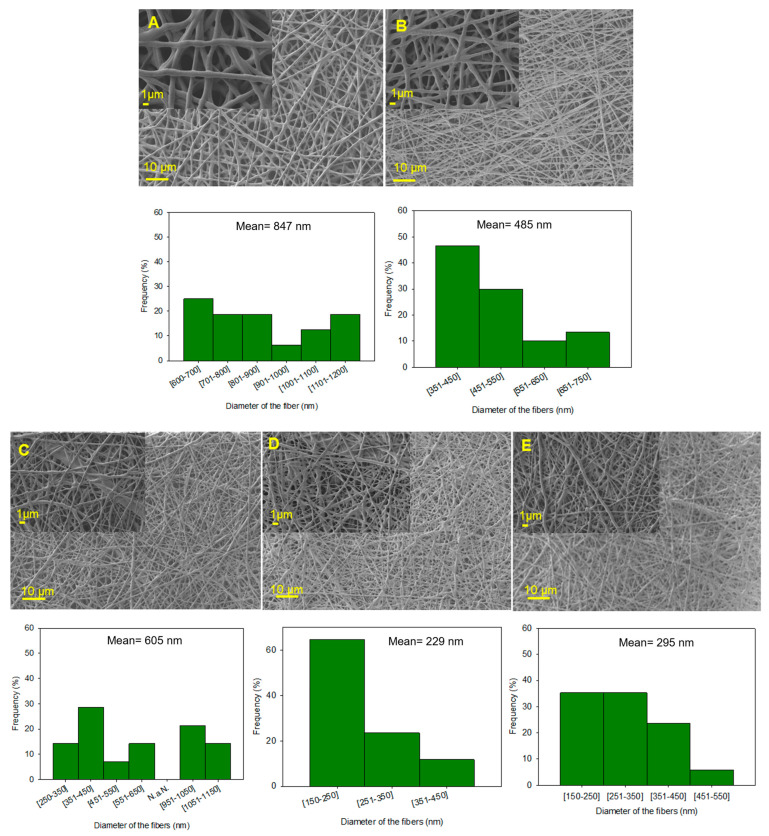
SEM micrographs of the electrospun membranes with a magnification of ×1000 and ×5000 (insert) and their distribution by size (on bottom). (**A**)—membrane of PEA_ala_100k; (**B**)—membrane of PEA_phe_50k; (**C**)—membrane of PEA_ala_50k and PEA_phe_50k (50:50); (**D**)—membrane of PEA_ala_50k and PEA_phe_50k (75:25); (**E**)—membrane of PEA_ala_50k and PEA_phe_50k (25:75).

The morphology of the membranes and the fiber diameter distribution were evaluated by SEM (Figure 4). In terms of morphology, the membranes presented well-defined and individualized fibers, almost free of structural defects. The PEA_ala_50k/PEA_phe_50k membranes obtained from the 75:25 and 25:75 blends showed more defects, but still individualized fibers, a result that can be attributed to the fact that two polymers with different structures are being electrospun at the same time. The membrane prepared using PEA_ala_100k presented the higher value for fibers’ diameter (average diameter of 847 nm) and a broader size distribution. On the other hand, the PEA_phe_50k membranes exhibited a narrower size distribution and lower fiber diameter values (average diameter of 485 nm). For the membranes with the same weight ratio of PEA_ala_50k and PEA_phe_50k, the width of the size distribution increased, which may be a result of two different polymers being electrospun simultaneously [38,50]. As a consequence, the average fiber diameter presented values of about 605 nm. This was not observed with the other membranes based on PEA_ala_50k and PEA_phe_50k, whose average diameter was in the range 200–300 nm.

**Figure 5:**
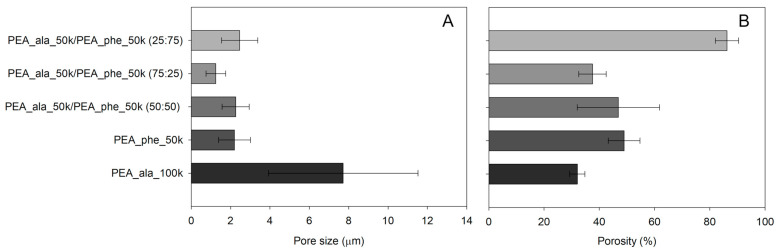
Pore size (**A**) and porosity (**B**) of the AAA-PEA-based membranes.

In the prevention of tendon adhesions, one of the most important properties that the membranes should possess is their ability to avoid the infiltration of exogenous fibroblasts into the injured site. Consequently, if the pore size of the membranes is smaller than the diameter of the fibroblasts, cell infiltration into the membrane is hindered. In their round form, fibroblasts possess a diameter of 8–10 µm, while in their elongated form, they have a diameter of about 50 µm [3,16]. Pore size (Figure 5A) was evaluated by SEM, and it should be noted that only pores present on the surface of the membranes can be measured. The membranes prepared with PEA_phe_50k alone and from blends of PEA_ala_50k and PEA_phe_50k have surface pores with a diameter smaller than 8 µm, which in principle would prevent the infiltration of exogenous fibroblasts into the injury site. The pore size values determined for PEA_ala_100k membranes were higher and more dispersed, but the mean value is around 8 μm and therefore should also be able to prevent fibroblast infiltration.

The porosity of the membranes was also determined, considering the density of the polymers that compose the membrane and the volume occupied by a given mass of the membrane, as previously described [3]. The results are presented in Figure 5B. The membranes prepared using the PEA_ala_50k/PEA_phe_50k (75:25) blend presented the lowest porosity value, so it could be representative of a more entangled network. In fact, this membrane has most of the fibers in a diameter range of [150–250] nm, which allows better fiber stacking and, thus, reduced porosity. The membrane made from PEA_phe_50k presented the highest porosity. In this case, the distribution of fiber diameter is very broad; therefore, there is more difficulty in packing the fibers, which might lead to an increase in the number of pores in the membrane structure.

#### 3.2.2. Water Uptake of the Membranes

**Figure 6:**
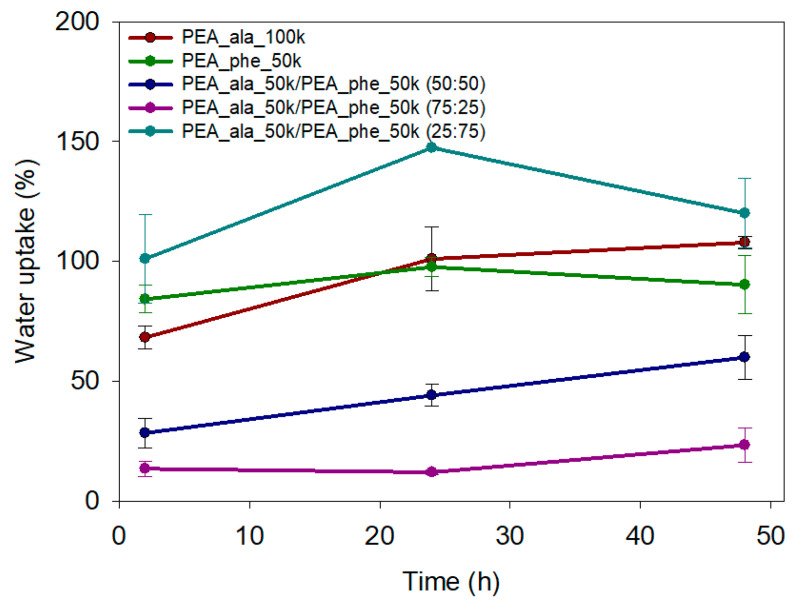
Water uptake of the prepared membranes.

The membranes were characterized in terms of their water uptake by immersion in a solution of PBS (pH = 7.4), at 37 °C. The results can be seen at Figure 6.

Figure 6 shows that PEA_ala_100k and PEA_phe_50k have similar swelling capacity values. It was expected that the membranes prepared from PEA_phe_50k would have a lower swelling capacity, since L-phenylalanine is more hydrophobic than L-alanine. This result can be attributed to the different porosity of the membranes; compared to the PEA_ala_100k membrane, the PEA_phe_50k membrane has a higher porosity, so that the expected lower affinity of this membrane to the aqueous medium is compensated by its higher porosity, which allows the absorption of a larger amount of the swelling medium.

The membranes obtained from the blends of PEA_ala_50k and PEA_phe_50k show that the one with the higher swelling capacity is also the one with the higher porosity (PEA_ala_50k/PEA_phe_50k (25:75)). Conversely, the membrane with the lower swelling capacity is the one with the lowest porosity (PEA_ala_50k/PEA_phe_50k (75:25)).

For the PEA_phe_50k and (PEA_ala_50k/PEA_phe_50k (25:75)) membranes, a decrease in swelling capacity is observed after the maximum is reached. This behavior can tentatively be attributed to the release of some residues from the membrane, which leach into the swelling medium. Among the membranes based on a single AAA-PEA (PEA_phe_50k vs. PEA_ala_100k) or those in blends, the PEA_phe_50k and PEA_ala_50k/PEA_phe_50k (25:75) membranes exhibit the highest porosity, making them more prone to leaching out some residues.

The results obtained, therefore, indicate that the swelling capacity of the membranes is determined by their porosity and not by the intrinsic hydrophilicity of the AAA-PEAs from which the membranes are prepared.

The PEA_ala_50k/PEA_phe_50k (75:25) membrane exhibited the lowest water uptake value, which is unsuitable for the intended application, as it could compromise the lubrication properties of the membrane. For this reason, this membrane was not considered for subsequent testing.

#### 3.2.3. Permeability of the Membranes

**Figure 7:**
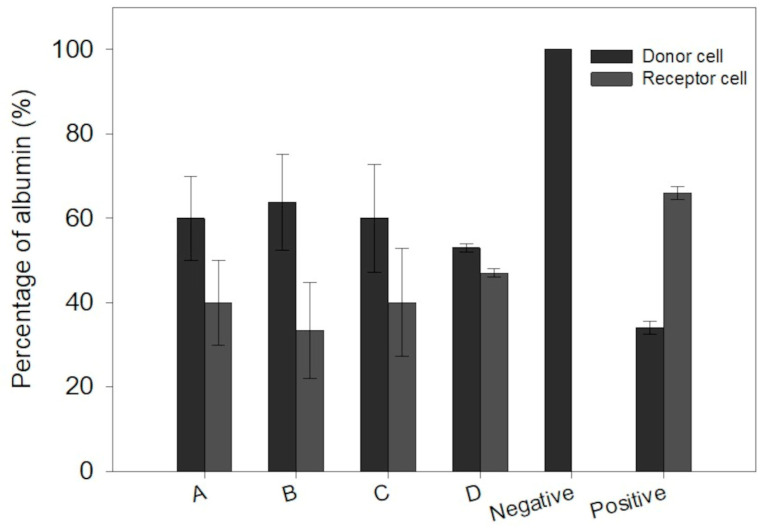
Percentage of albumin found in the donor and receptor cell after one week of testing.

Considering the intended application of the membranes, it is important that the membranes allow the flow of nutrients and growth factors in order to not compromise the intrinsic healing of the tendon. To ensure this, a permeability test was performed using albumin (see Supplementary Information, Figure S3), as it is the most abundant protein in synovial fluid [51]. The donor cell had an initial albumin concentration of 1.4 mg/mL. The permeability of the membranes was determined after one week. The absorbance of each sample in the receptor and donor cell was measured, and the value was then extrapolated to the mass of albumin present in each flask. This value was adjusted according to the volume in each flask and the results are expressed as a percentage compared to the total mass of protein. A positive (open flask) and negative (closed flask) control were used to ensure the viability of the values (Figure 7).

In Figure 7, it is possible to confirm the presence of albumin in all the receptor and donor cells (with the exception of the negative control), indicating that all membranes allowed the passage of the protein. The PEA_ala_50k/PEA_phe_50k (25:75) membrane is the one that allows the passage of more albumin to the receptor cell, which is due to its high swelling capacity compared to the other membranes.

#### 3.2.4. In Vitro Enzymatic Degradation

As previously mentioned, the degradation of AAA-PEAs could be enhanced by proteases such as α-chymotrypsin and lipase, depending on the α-amino acid used [34]. These two proteases were selected for the enzymatic degradation study because they are overexpressed in the inflammatory process associated with tendon injury [21].

**Figure 8:**
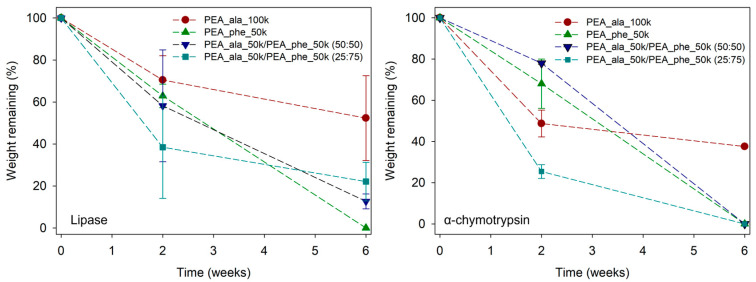
Percentage of remaining weight of each membrane after degradation in an enzymatic medium using lipase and α-chymotrypsin, for a period of 6 weeks.

Lipase is responsible for the hydrolysis of ester bonds [48,52]. The more pronounced degradation of the PEA_ala_50k/PEA_phe_50k (50:50) membrane compared to the PEA_ala_50k/PEA_phe_50k (25:75) membrane (Figure 8) could be due to the fact that the latter has fewer bulky groups in its structure, making it easier for the enzyme to reach the linkage to be degraded [21]. However, when looking at the weight loss of membranes made of PEA_ala_100k and PEA_phe_50k (Figure 8), it can be seen that the former has a lower weight loss, which could be a direct consequence of the higher molecular weight of PEA_ala compared to PEA-phe. It should be noted that, with the exception of the PEA_phe_50k membrane, all membranes maintained their integrity after 6 weeks of testing when exposed to the action of lipase.

Regarding the degradation mediated by α-chymotrypsin, it has been reported that this enzyme has a preference for amide linkages next to hydrophobic α-amino acids and in particular for L-phenylalanine [30,31,45,52]. Degradation studies with α-chymotrypsin have also been reported on electrospun membranes from PEA_phe, which showed a weight loss of almost 80% weight loss after 6 days [45] and 100% after 14 days [40]. After 6 weeks, all membranes, with the exception of PEA_ala_100k, reached a residual weight value close to zero (Figure 8). For the membranes from the blends, a higher percentage of PEA_phe_50k could allow a better interaction of the enzyme with the polymer chains and enhance the effect of the enzyme on the degradation of the amide bond.

#### 3.2.5. Mechanical Properties

**Figure 9:**
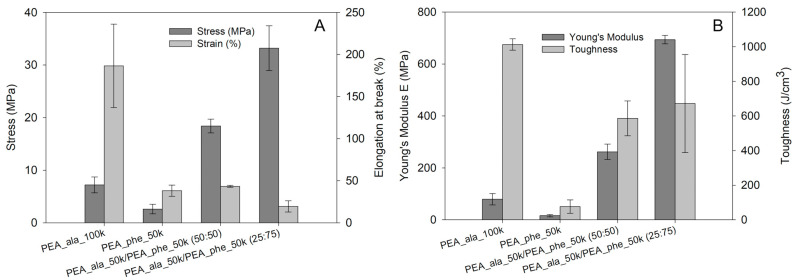
Mechanical properties of the membranes ultimate tensile strength and elongation at break (**A**), Young’s modulus and toughness (**B**).

The mechanical properties of membranes provide information about their suitability for the intended application, showing their response to various mechanical forces. In this case, tensile tests were performed. The results are summarized in Figure 9 and Table S2 (see Supplementary Information). The ability of the material to withstand forces can be seen from the tensile strength or maximum stress [53]. In Figure 9A, it is possible to observe that the membrane prepared using only PEA_ala_100k or the blend with PEA_ala_50k/PEA_phe_50k (25:75) presented the highest ultimate tensile strength at rupture, with the first one also showing the highest value for elongation at break. This result might be related to the larger fiber diameter of these membranes. For the envisaged application, the membranes should be able to withstand loads, as the tendon is subjected to multiple forces, but also be stretchable enough to be placed in situ. So, in this case, the membrane made of PEA_phe_50k presented modest values, having the worst mechanical performance. For the other properties, such as Young’s modulus and toughness, the membrane made from the PEA_ala_50k/PEA_phe_50k (25:75) blend continued to present higher values. This means that this particular membrane has a higher resistance to stretching or tensile forces without breaking, as it is able to absorb more energy per unit of volume until the rupture of the material [54].

#### 3.2.6. Thermomechanical Properties of the Membranes

**Figure 10:**
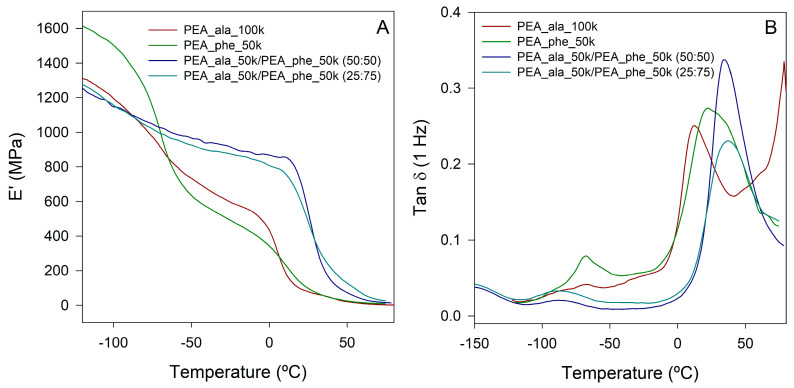
DMTA traces of the membranes at the frequency of 1 Hz; (**A**)—E vs. T’; (**B**)—Tan δ vs. T.

The dynamic mechanical properties of the prepared membranes were assessed by DMTA, in multifrequency mode, and the results can be found in Figure 10 and Table 3. The transition from the glassy to the rubbery state of the membranes takes place in a narrower temperature range for the membranes prepared from the blends than for the membranes prepared from a single AAA-PEA. Also, this transition occurs at higher temperatures for the membranes prepared from the blends (Figure 10A). In the tan δ vs. *T* profile (Figure 10B), two transitions can be distinguished; the first occurs in a temperature range consistent with a β-transition and should correspond to the local motions of methyl and aromatic groups in the structure of the AAA-PEAs. This transition occurs at different temperatures for membranes prepared from a single polymer and for membranes prepared from blends, being lower for the latter. This observation suggests that the presence of two chemically distinct pendant groups in the membranes facilitates enhanced mobility of each group, presumably due to the disruption of interactions between groups of the same chemical nature. As for the *T*_g_ values, it is possible to see that the membranes of the blends present a higher *T*_g_ value than those prepared from a single polymer, which is somewhat unexpected. Typically, in a blend, if the polymers that compose the blend are miscible, a single *T*_g_ is observed, and the value should be between those of the individual polymers. Perhaps, during the electrospinning process, stronger interactions between the main polymer chain are established, which restrict the movement of the chain, leading to an increase in the *T*_g_ value. It can also be seen from the tan δ vs. *T* profile that the PEA_ala_50k/PEA_phe_50k (50:50) membrane has the highest value of tan δ, suggesting a greater capacity for energy dissipation. This behavior could contribute to improved membrane performance under mechanical stress. As for the E’ values, in the temperature range from room temperature (about 25 °C) to body temperature (about 37 °C), it can be seen that the membranes based on the blends have higher values than the membranes based on PEA_ala or PEA_phe. These results are consistent with those from the tensile tests and may be attributed to the possible interactions established between the two AAA-PEAs. In fact, the membranes based on the blends have higher values of E’ than the PEA_ala_100k, which is the membrane that has the fibers with the largest diameter and the lowest porosity, two characteristics typically associated with improved mechanical properties in electrospun membranes.

#### 3.2.7. In Vitro Biological Evaluation

**Figure 11:**
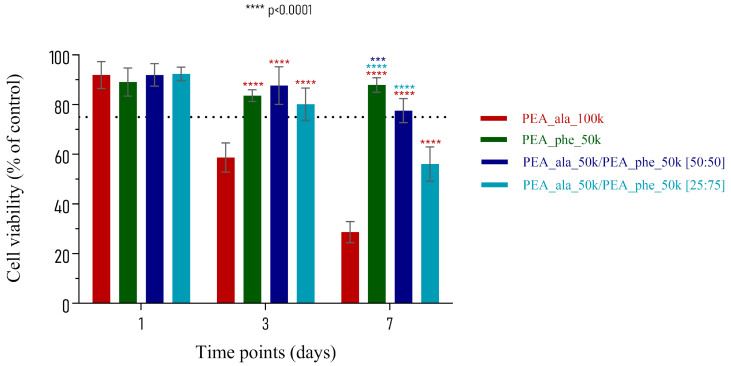
Metabolic assay of NIH3T3 fibroblasts cultured on the prepared electrospun membranes during 1, 3 and 7 days. Results are expressed as the mean of three independent experiments represented with the standard error. Statistical significance levels were set at *** *p* = 0.0002 and **** *p* < 0.0001.

To assess the interaction between the membranes and fibroblasts, these cells were seeded directly on the membrane surface. The cell proliferation was measured through the metabolic activity of NIH3T3 cells using the AlamarBlue™ HS Cell Viability reagent. In Figure 11, it is possible to see the percentage of viable cells for 1, 3 and 7 days.

**Figure 12:**
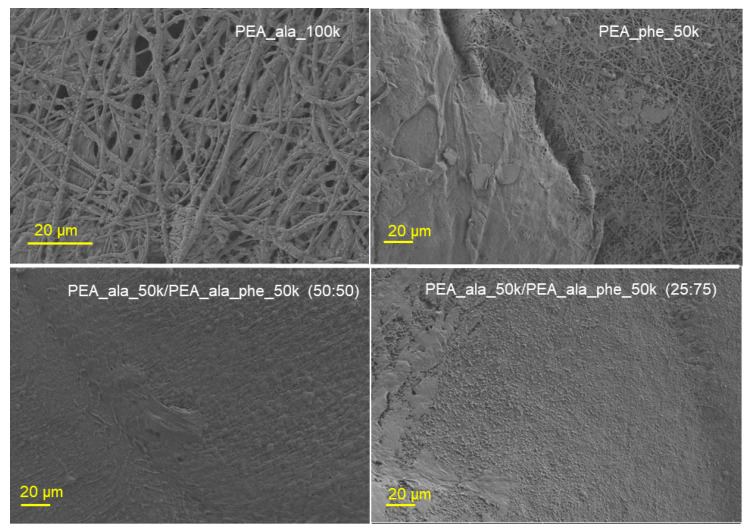
SEM micrographs of the morphology of NIH3T3 cells seeded into the electrospun membranes after 3 days at a magnification of ×1000 (PEA_ala_100k) and ×500.

On the first day, there is almost no significant difference between the viability observed for all membranes. However, after 3 days, the number of viable cells in contact with the membrane made of PEA_ala_100k (58.730 ± 5.874%) is significantly lower when compared to the other membranes. The value presented is less than 75%, representing some degree of cytotoxicity. After 7 days, cell viability is also reduced for the membrane prepared with the PEA_ala_50k/PEA_phe_50k (25:75) blend (56.100 ± 6.924%). It was hypothesized that this reduction in the cell viability could be related with some anti-adhesive effect of the membranes towards fibroblasts [40,55]. Thus, to verify if this was the case, the membranes were analyzed by SEM. As we can see from Figure 12, although some cells are still present, their tendency, after 3 days, is to change from an elongated shape to a rounder shape, indicating their inability to adhere properly. In fact, the membrane that had the lower viability (PEA_ala_100k) has almost no cells at the surface (Figure 12).

The membranes made of PEA_phe_50k and the PEA_ala_50k/PEA_phe_50k (50:50) blend proved to be non-cytotoxic at all time points studied. After 7 days, the membranes made of PEA_ala_100k (28.721 ± 4.270%) and the PEA_ala_50k/PEA_phe_50k (25:75) blend (56.100 ± 6.924%) presented reduced viability, indicating lower cytocompatibility that could be related to the higher anti-adhesion effect produced.

## 4. Conclusions

In this study, L-alanine- and L-phenylalanine-based PEAs were successfully used in the prevention of tendon adhesions. Given that the electrospinning of PEAs, particularly AAA-PEAs, is not well explored, this research combines the innovative use of these polymers in the electrospinning process with their application in preventing tendon adhesions. For PEA_ala, a molecular weight of approximately 100 kDa was necessary to obtain membranes with individualized and defect-free fibers. In contrast, a molecular weight of 50 kDa was enough for PEA_phe. Using PEA_ala and PEA_phe with comparable molecular weights (50 kDa), three blends were prepared (PEA_ala_50k/PEA_phe_50k (50:50), PEA_ala_50k/PEA_phe_50k (25:75), and PEA_ala_50k/PEA_phe_50k (75:25)) and subjected to the electrospinning process. The PEA_ala_50k/PEA_phe_50k (75:25) membrane presented a low water uptake value and was consequently excluded from further testing. All selected membranes exhibited permeability to albumin, suggesting that the membranes would permit the passage of nutrients essential for tendon healing. Conversely, the membranes demonstrated pore size values suitable for impeding the passage of fibroblasts. The membrane composed of PEA_ala_100k and the PEA_ala_50k/PEA_phe_50k (25:75) blend demonstrated the highest resistance to tensile deformation. The reduced cell viability observed for these two membranes after 7 days suggests a lack of cell adhesion, likely attributable to the presence of a non-fouling surface. This characteristic is crucial for the intended application, as it inhibits cell attachment and subsequent adverse biological responses. Among all tested membranes, the PEA_ala_50k/PEA_phe_50k (25:75) blend offered the best compromise between the various properties, emerging as the most promising membrane for the envisaged application.

## Figures and Tables

**Table 1:** Amounts of AAA-PEAs and TFE used in the solutions for ES, and respective final concentration.

Formulations	Weight Ratio	m_PEAs_ (g)	V_TFE_ (mL)	Final Concentration (% *m*/*v*)
PEA_ala_100k	--	0.6	2	30
PEA_phe_50k	--	0.6	2	30
PEA_ala_50k/PEA_phe_50k	50:50	0.2/0.2	2	20
	25:75	0.1/0.3	2	20
	75:25	0.3/0.1	2	20

**Table 2:** Thermal properties obtained from the TGA and DSC analysis: *T*_5%_—temperature at which the samples lose 5% of their initial weight; *T*_onset_—onset temperature; *T*_g_—glass transition temperature; *T*_m_—melting temperature; Δ*H*_m_—melting enthalpy.

	*T*_5%_ (°C)	*T*_onset_ (°C)	*T*_m_ (°C)	Δ*H*_m_ (J/g)	*T*_g_ (Cooling)
PEA_ala_50k	326	336	89/108	13/4.7	15.2
PEA_ala_100k	308	343	104	32.5	18.6
PEA_phe_50k	280	295	112	38	27.4

**Table 3:** Values obtained for the electrospun membranes by DMTA.

	*T*_β_ (°C)	*T*_g_ (°C)	E′_25°C_ (MPa)	E′_37°C_ (MPa)
PEA_ala_100k	−67	12.2	78	51
PEA_phe_50k	−67.5	22.3	97	49
PEA_ala_50k/PEA_phe_50k (50:50)	−88.8	34.4	539	177
PEA_ala_50k/PEA_phe_50k (25:75)	−88.4	37.2	482	245

## Data Availability

Data is contained within the article or Supplementary Material.

## Supplementary Materials

The following supplementary information can be downloaded at: https://www.mdpi.com/article/10.3390/polym17030395/s1, Figure S1. ^1^H NMR spectra of the the di-p-toluenesulfonic acid salts of the bis-α-(L-amino acid)-α,ω-alkylene diesters from L-alanine (A) or L-phenylalanine (B) and 1,6-hexanediol. Figure S2. ^1^H NMR spectra of the synthesis of the activated diester from sebacoyl chloride. Figure S3. Permeability apparatus. Figure S4. Calibration curve used for the permeability tests. Figure S5. TGA and DTG curves of the synthetized PEA_ala_100k (A) and the heat flow curve of the same PEA obtained by DSC in the 1st heating (B). Figure S6. Heat flow of the synthetized PEAs in the second heating and cooling. Table S1. Optimization process of the synthetized membranes. Table S2. Tensile strength, elongation at break, elastic modulus and toughness of the electrospun membranes.
