# Supplementary material for: Tackling the Problem of Tendon Adhesions: Physical Barriers Prepared from α-Amino Acid-Based Poly(ester amide)s
Source: Polymers (Basel). 2025 Feb 1;17(3):395. doi: 10.3390/polym17030395 (PMC11821251; doi:10.3390/polym17030395)
Supplement: Supplementary file 1 [file polymers-17-00395-s001.zip › polymers-3386877-supplementary.pdf]

Supplementary information for:

Tackling the Problem of Tendon Adhesions: Physical Barriers Prepared from α-Amino Acid- Based Poly(ester amide)s

Sofia Saraiva[1], Francisca Rénio[1], Patrícia Pereira[1,2], Patrícia Santos[1], Carlos T. B. Paula[1,2], Amílcar Ramalho[3], Arménio C. Serra[1], Ana C. Fonseca[1*]

[1]Centre for Mechanical Engineering, Materials and Processes (CEMMPRE), ARISE, Department of Chemical Engineering, University of Coimbra, Pólo II, Rua Sílvio Lima, 3030-790 Coimbra,

[2] IPN, Instituto Pedro Nunes, Associação para a Inovação e Desenvolvimento em Ciência e Tecnologia, Rua Pedro Nunes, 3030-199 Coimbra, Portugal

[3]Centre for Mechanical Engineering, Materials and Processes (CEMMPRE), ARISE, Department of Mechanical Engineering, University of Coimbra, Pólo II, Rua Luís Reis Santos, 3030-788 Coimbra, Portugal

*Correspondence: anafs@eq.uc.pt

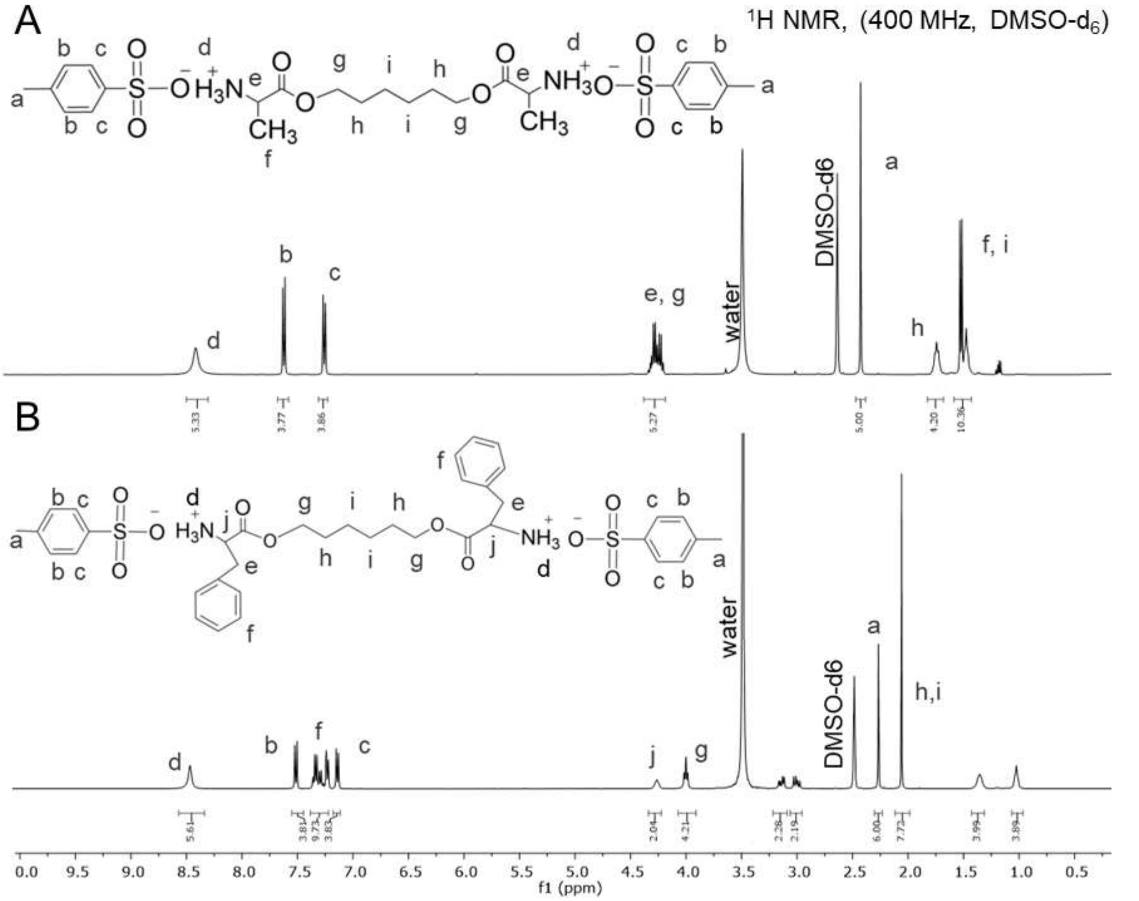

Figure S1. ^{1}H NMR spectra of the the di-p-toluenesulfonic acid salts of the bis-α-(L-amino acid)-α,ω-alkylene diesters from L-alanine (A) or L-phenylalanine (B) and 1,6-hexanediol.

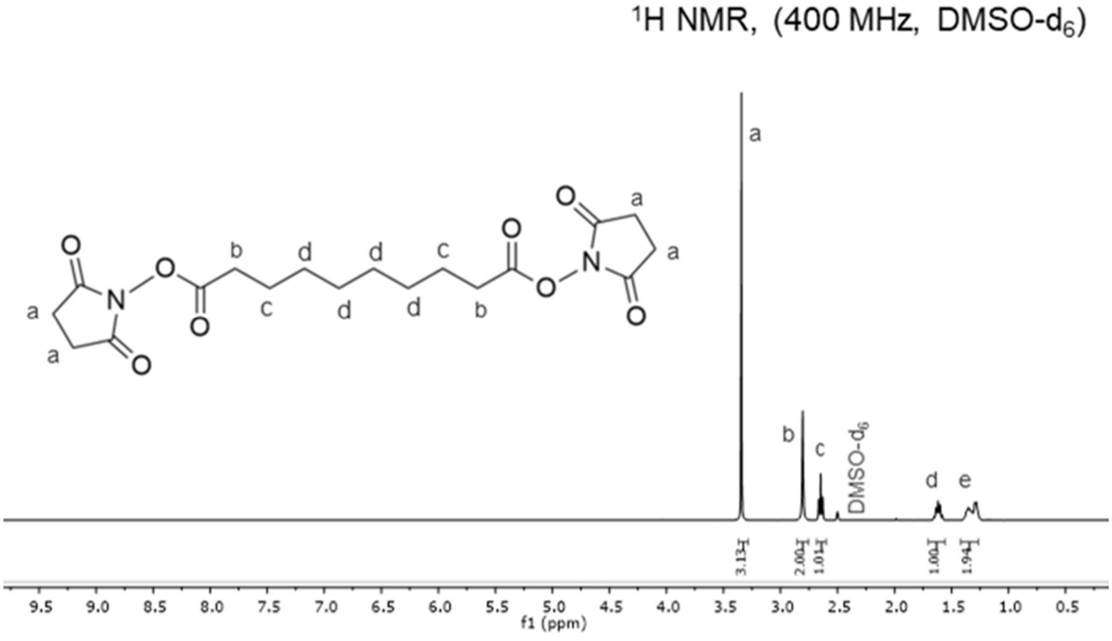

Figure S2. ^{1}H NMR spectra of the Synthesis of the activated diester from sebacoyl chloride.

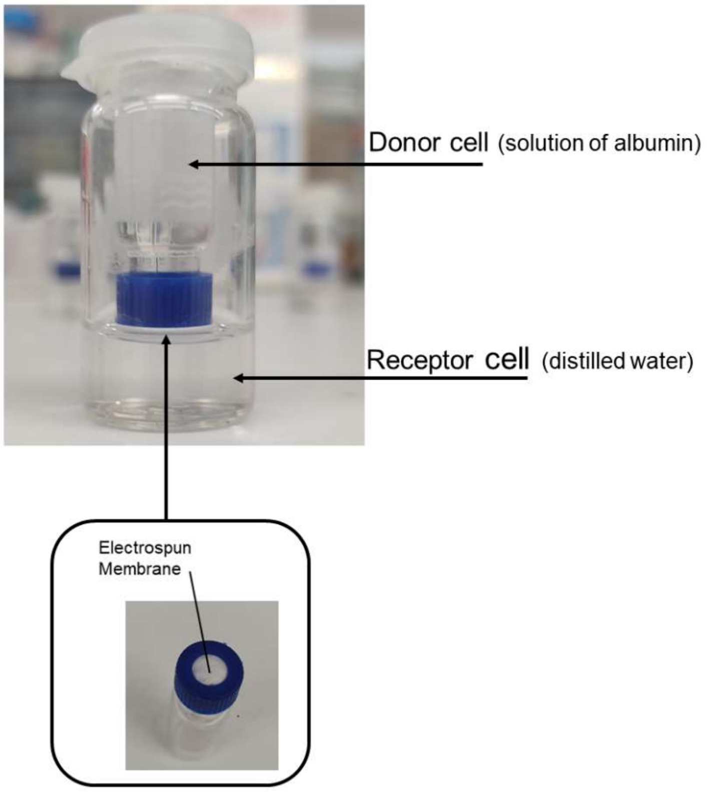

Figure S3. Permeability apparatus.

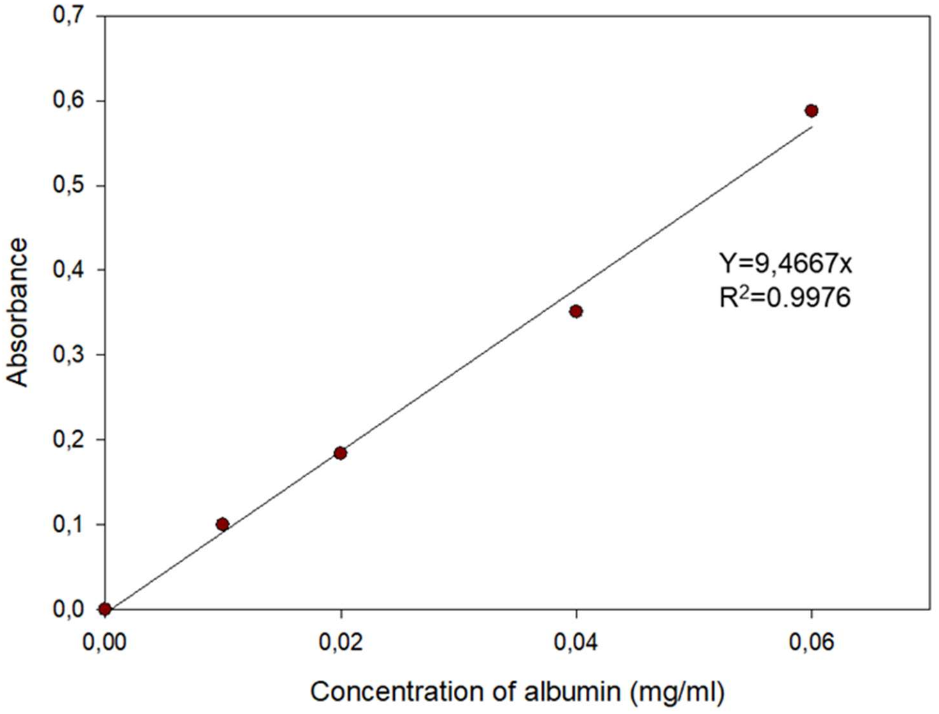

Figure S4. Calibration curve used for the permeability tests.

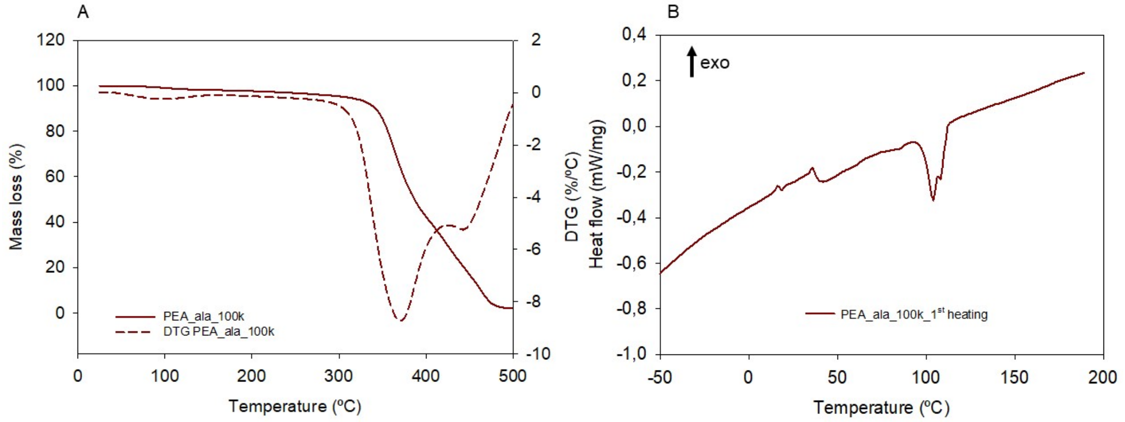

Figure S5. TGA and DTG curves of the PEA_ala_100k synthetized (A) and the heat flow curve of the same PEA obtained by DSC in the 1st heating (B).

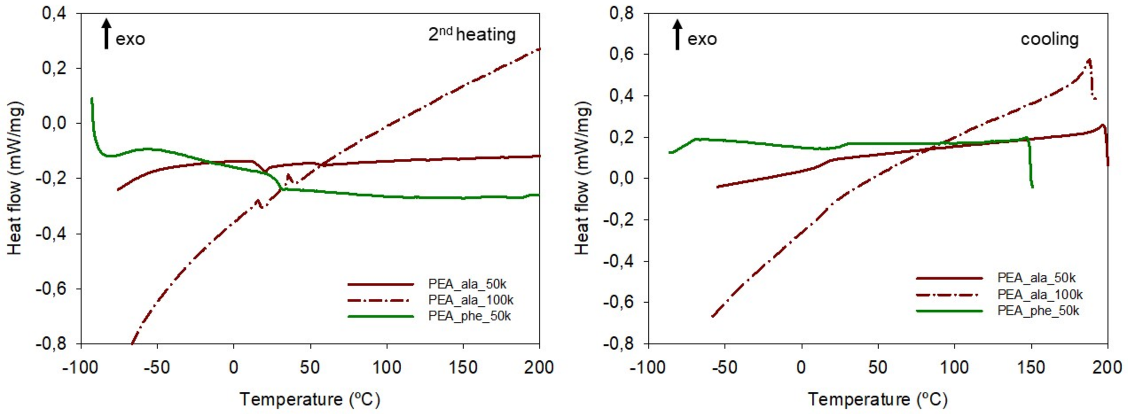

Figure S6. Heat flow of the synthetized PEAs in the second heating and cooling.

Table S1. Different parameters changed in the optimization process of the prepared membranes.

Polymer	Solvent	Concentration (%w/v)	Voltage (kV)	Collector distance (cm)	Flow rate (mL/h)	Humidity (%)	Result
**PEA_ala_50K**	TFE	50	20	20	0.5 mL/hr	40	Deposition in powder
	TFE	50	16-17	15	0.8 mL/hr	20	Thick and unflexible membrane
	TFE	20	17	10	0.5 mL/hr	20	Membrane (wout/ defined fibers in SEM)
**PEA_ala_100k**	TFE	35	16	12	0.5 mL/hr	20	Membrane with defects
	TFE	30	17	12	0.5 mL/hr	20	Membrane with defects
**PEA_phe_50k**	TFE	25	24	12	0.5 ml/hr	20	Membrane (wout/ defined fibers in SEM
	TFE	15	15.5	12	0.5 ml/hr	20	Membrane (wout/ defined fibers in SEM
	TFE	30	17	15	0.5 ml/hr	20	Membrane with defects
**PEA_ala_50k/ PEA_phe_50k (50:50)**	TFE	20	14	12	0.5 mL/hr	30	Membrane with defects
	TFE	20	15	12	0.5 mL/hr	30	Membrane with defects
	TFE	20	16	12	0.5 mL/hr	30	Membrane with defects
	TFE	20	15.5	12	0.5 mL/hr	30	Membrane with defects
	TFE	20	17	12	0.5 mL/hr	30	Membrane with defects
**PEA_ala_50k/ PEA_phe_50k (75:25)**	TFE	20	15.5	12	0.5 mL/hr	30	Membrane with defects
**PEA_ala_50k/ PEA_phe_50k (25:75)**	TFE	20	15	12	0.5 mL/hr	30	Membrane with defects
	TFE	20	17	12	0.5 mL/hr	30	Membrane with defects

Table S2. Tensile strength, elongation at break, elastic modulus and toughness of the electrospun membranes.

	Tensile strength (MPa)	ε (%)	E (MPa)	Toughness (J/cm^3)
**PEA_ala_100k**	7.2 ±1.51	186.44 ±49.56	79.38±21.83	1012.64±49.82
**PEA_ala_50k**	2.61±0.92	38.17±6.69	15.99±4.77	76.46±38.58
**PEA_ala_50k/PEA_phe_50k (50:50)**	18.42±1.30	43.34±1.17	261.94±29.36	586.02±100.38
**PEA_ala_50k/PEA_phe_50k (25:75)**	33.21±4.25	19.46±5.45	693.87±16.23	671.7±282.99
